# The Global Burden of Rheumatic Heart Disease: Population-Related Differences (It is Not All the Same!)

**DOI:** 10.21470/1678-9741-2020-0514

**Published:** 2020

**Authors:** Manuel J. Antunes

**Affiliations:** 1 Clinic of Cardiothoracic Surgery, Faculty of Medicine, University of Coimbra, Coimbra, Portugal.

**Keywords:** Rheumatic Heart Disease, Mitral Valve, Mitral Valve Repair, Developing Countries, Altruism

## Abstract

Rheumatic heart disease (RHD) remains the most common cardiovascular disease in young adults and adolescents in need of heart surgery in low- and middle-income countries (LMICs). The mean age of patients is 20-25 years, often much younger. By contrast, the few patients with chronic RHD in developed countries present a mean age of around 55 years. It is absolutely fundamental to differentiate these two types of population. Pathology, lesions and surgical methods are different, and the results should not be compared. It is not all the same!

A certain enthusiasm for mitral repair has recently surged, with several reports showing excellent results in children and young adults, resulting from the renewed interest of cardiac surgeons, also based on new and modified techniques developed in the meantime.

While surgery is easily accessible to patients in developed countries, the situation in LMICs is often dramatic, with countries where there is a complete absence of or few surgical facilities absolutely unable to meet gigantic demands. Many foreign surgical teams conduct humanitarian missions in several of these countries. They are just a “drop of water in the ocean” of needs. In some cases, however, these missions led to the establishment of local teams that now work independently and, in some cases, outperform the foreign teams still visiting.

**Table:** 

Abbreviations, acronyms & symbols	
ARF	= Acute rheumatic fever
GDP	= Gross domestic product
LMICs	= Low and middle-income countries
MVREP	= Mitral valve repair
PTFE	= Polytetrafluoroethylene
RF	= Rheumatic fever
RHD	= Rheumatic heart disease
SUS	= Brazilian national health system

## INTRODUCTION

Rheumatic fever (RF) is still endemic in several regions of the world, especially in low- and middle-income countries (LMICs) of the Southern Hemisphere and in some areas of Asia, where it remains one of the most important causes of premature death. Acute rheumatic fever (ARF) mainly affects children aged 5 to 14 years, but recurrent episodes of ARF remain relatively common in adolescents and young adults, up to the age of 30 to 40 years. The real incidence of RF in these countries is still unknown, as it is largely a neglected and underdiagnosed non-communicable disease. On the other hand, the developed countries of the Northern Hemisphere, where the disease was still prevalent in the 50s and 60s of the 20^th^ century, are now virtually free from new cases.

**Fig. 1:**
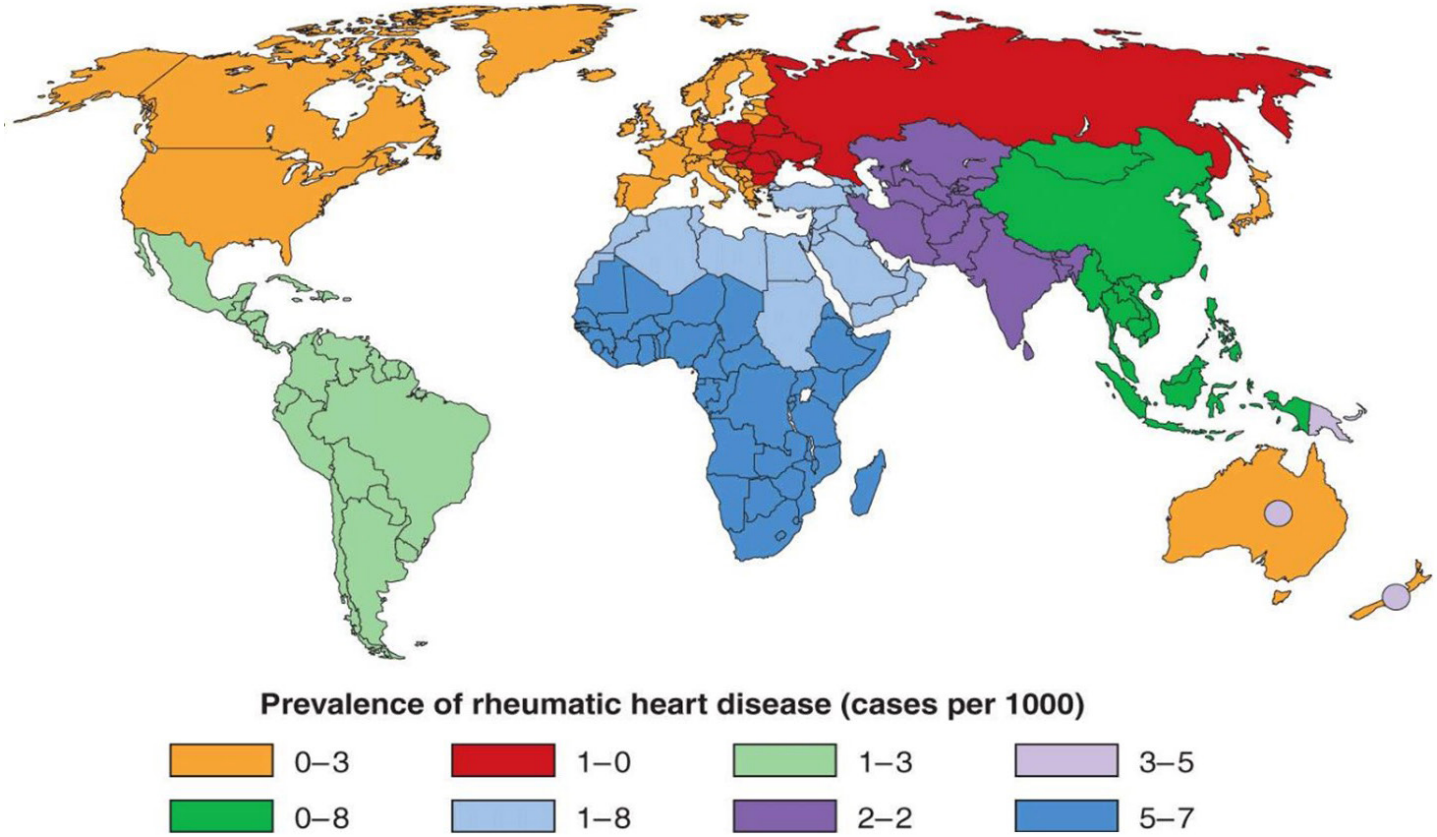
Prevalence of rheumatic heart disease in children aged 5 to 14 years (adapted from Carapetis et al.^[2]^).

Up to 60% of patients with ARF progress to rheumatic heart disease (RHD) (Figure 1). It is estimated that there are still over 33 million cases of RHD worldwide and 300,000 deaths in 2015^[1]^. Figure 1 shows the global prevalence of the disease, which affects mainly the valves, especially mitral and aortic. It is an inflammatory process that produces scarring and distortion of the valve apparatus and causes either regurgitation or stenosis, or both, which leads to surgery in most cases. The degree and speed of valve involvement varies greatly, depending on the effectiveness of RF control. In LMICs, the lack or insufficiency of medical care facilitates a fast progression of the disease that requires surgery, when available, much earlier. This is no longer a problem in developed countries, which, however, still have many patients with chronic remnants of the acute disease acquired in their early years of life.

RHD remains the single most common cardiovascular disease in young adult and adolescent patients in need of heart surgery, outweighing other indications such as congenital cardiac defects by almost 4-fold^[3,4]^. The mean age of patients submitted to surgery is around 20-25 years, half of whom require operation under the age of 20, some even under the age of 10^[5]^. By contrast, the fewer patients with chronic RHD from developed countries present to surgery at a mean age of around 55 years. These differences have an impact on the valve morpho-pathology observed, with consequences in the type of treatment applied. Naturally, these are two different types of pathology, and the reported series should not be compared, as discussed below.

The current situation in Brazil appears to be in transition between the two epidemiological phases described above, with a significant decrease in new cases of RHD in the last decade, at least in the larger urban centres, which will undoubtedly follow the predominance of late occurring forms of pathology. But the prevalence of RF is still high in rural areas, where access to medical care is less uniformly available. It was recently estimated that approximately 37,500 new patients per year developed chronic RHD in the period 2014-2018 in Brazil^[6]^.

However, it is important to emphasize that, in the majority of countries, the prevalence of RF and RHD is underestimated because it is calculated on the basis of partial and sectorial observations rather than on large systematic and comprehensive epidemiological studies. Efforts to address the disease at its root are urgent and necessary. Early detection of ARF and RHD through screening programs can reduce the morbidity of chronic RHD and the number of surgeries required. Systematic echocardiographic examinations have consistently increased the rates of diagnosed RHD^[7]^. Of note, the cost of a single surgical intervention for RHD, varying from US$10,000 to US$25,000, would be enough to fund 1 year of an RHD screening program in a LMIC.

### Pathophysiology

**Fig. 2:**
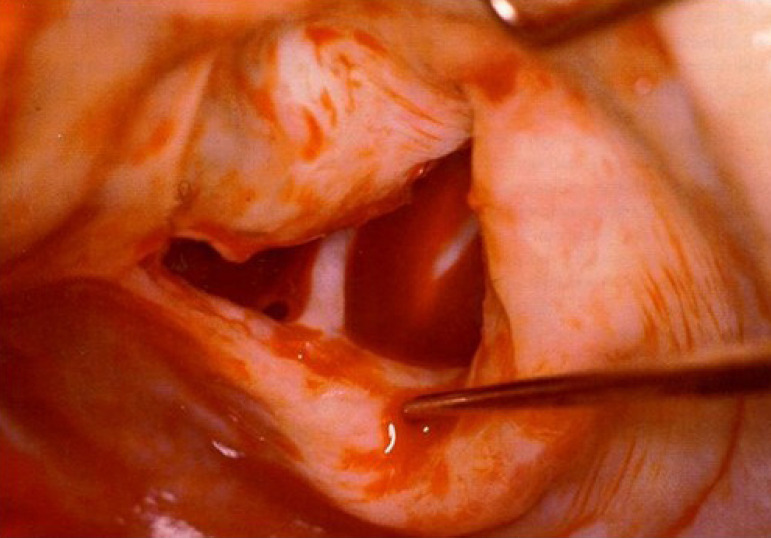
Typical acute rheumatic mitral valve of a 10-year old child, with inflamed and thickened leaflets and partially fused commissures, submitted to repair.

The pathophysiology of RHD is still not well understood^[8]^. Why are there cases that evolve very quickly, often involving both left heart valves, requiring very early intervention, sometimes during the acute phase of the disease (Figure 2)? Once the valves are involved, the rheumatic process, which may be of both inflammatory and metabolic nature, is almost unstoppable and leads to further inflammation and scarring of the valve tissue. By contrast, in other patients the disease evolves much more slowly, entering a chronic process that may need treatment only at a much older age. Also, it remains unknown why valve insufficiency occurs in some patients (the majority), while in others it evolves predominantly to stenosis.

It is often said that mitral valve stenosis manifests itself at a very late and chronic stage. However, pure mitral stenosis, caused by fusion of the commissures, with almost normal leaflets, may appear in patients in the second and third decade of life; I have treated 15-year olds with pure, pliable mitral stenosis. Naturally, valve insufficiency and/or stenosis disturb the valve. Nevertheless, the predominant lesion in very young patients is mitral regurgitation, sometimes accompanied by a degree of aortic regurgitation. The hallmark of rheumatic mitral valve regurgitation in the young is prolapse of the anterior leaflet as a result of elongated chordae, predominantly in the centre of the leaflet (A2); occasionally, chordal rupture may be observed. Besides, the length of the leaflet is usually small for the size of the orifice. This is almost uniformly accompanied by annular enlargement and often by thickening and retraction of the posterior leaflet. Although annular dilatation occurs mainly at the expense of the posterior annulus, the anterior segment of the annulus may also enlarge (this is important in mitral valve repair; see below). Aortic valve regurgitation is usually caused by scarring and retraction of the cusps, and only rarely is the result of the annular aortic root dilatation. Aortic root dilatation is often present, as a result of regurgitation, and contributes to diminish cusp coaptation, further increasing regurgitation, in a vicious circle that can only be broken by valve repair or replacement.

### Role of Surgery

Once the valves become involved, there is no treatment that permits reversing the pathological changes, and chronic evolution of the lesions means that a significant number of patients with RHD will require surgery at some stage. However, the differences in presentation and pathology, as discussed above, oblige to different approaches. When reading the literature on surgery for RHD, it is absolutely fundamental to differentiate the two types of population described. Pathology, lesions and surgical methods are different, and the results should not be compared. We cannot compare a series of 419 children (≤18 years) from Malaysia or a series of 219 patients from São Paulo (mean age 27 years) with a series of 1,731 patients with a mean age of 52 years from South Korea^[9-11]^. It is not all the same!

Patients from LMICs are generally young, poor and uninstructed. They have difficult access to medical care and poor compliance to prophylaxis/therapy, with difficult followup. In these patients, the treatment should be based on two main considerations: valve repair *versus* replacement (especially of the mitral valve) and, in the latter, the use of mechanical or biological substitutes. Here, the occurrence of complications is more frequent; for example, the rates of thromboembolism and degradation of the bioprostheses are very high, as well as the incidence of infective endocarditis. On the other hand, recurrent RF episodes and the evolution of the rheumatic process places mitral valve repair (MVRep) at a greater risk of recurrence of regurgitation and/or stenosis^[12]^.

Roughly one third of the patients have aortic valve disease, either isolated or associated with mitral disease. Aortic regurgitation requires even more urgent surgery because of the greater negative impact on the left ventricle, with poor prognosis. However, repair of the aortic valve is in its infancy, although some have recently achieved good results with leaflet extension or mobilization/shaving techniques^[13,14]^. On the other hand, the rates of prosthetic-related complications are lower in the aortic valve than in the mitral valve position, both for mechanical valves and bioprostheses.

In the older patients, especially in developed countries, these considerations also pertain, but are somewhat less important as the sanitary conditions are much better and the rates of prosthetic complications are much lower. Hence, I will concentrate further discussion on surgery for RHD in the young.

### Mitral Valve Repair for RHD

The initial attempts to repair mitral valves quicky followed the first experiences of open heart surgery, in the 1950s and 60s, then motivated by the non-availability of valve substitutes, but the real advances were made by the Carpentier and Duran groups in the 1970s. Naturally, in those times, most cases were rheumatic, even in the after-war fast developing Europe and USA, and good results were initially reported by both groups and by others. However, as degenerative mitral valve diseases became more frequent, comparisons of surgery results for both pathological types were inevitable and rheumatic series mostly came in second, with worse long-term outcomes, especially in series predominantly with younger patients. However, MVRep almost always performed better in comparison to replacement, even in these circumstances^[10]^.

A degree of enthusiasm for valve repair in RHD has recently surged, with several publications from different parts of the world reporting excellent results in children and young adults^[9,15-19]^. This is a result of the renewed interest of cardiac surgeons, with increased experience, also based on the new and modified techniques in the meantime described. Here, I wish to emphasize the work of Dr. Kumar and his team in New Delhi, India, who, among others, have consistently been in the forefront of the efforts to promote rheumatic MVRep in children^[20]^.

However, the results of surgery are still dependent on controlling the rheumatic process and avoiding recurrence episodes of RF, in which penicillin prophylaxis is essential. Again, this is a problem in these populations because of poor compliance of the patients and shortage of medical services that can accomplish adequate postoperative long-term follow-up. It is a vicious circle almost impossible to control in most circumstances.

### Mitral Valve Repair Techniques in RHD

Better and newer repair techniques are made for significantly better results. In experienced hands, most of these valves (in my experience, over 75%) can be repaired with good results in the short and long term.

Carpentier’s famous “French correction”^[21]^ remained the basic concept for MVRep used by the large majority of surgeons engaged in this activity worldwide. In rheumatic cases, it was based on two surgical steps: chordal shortening and implantation of a rigid, complete ring. As discussed above, elongation of chordae with anterior leaflet prolapse and annular dilatation are the hallmarks of rheumatic mitral regurgitation. However, shortening of the chordae was found to be one of the weaknesses of the repair, because the chordae continued to elongate as the rheumatic process progressed. Artificial chordal substitution by polytetrafluoroethylene (PTFE) suture material has more recently proved to be much more consistent and durable^[22]^, hence it was rapidly adopted by a large number of surgeons around the world.

**Fig. 3:**
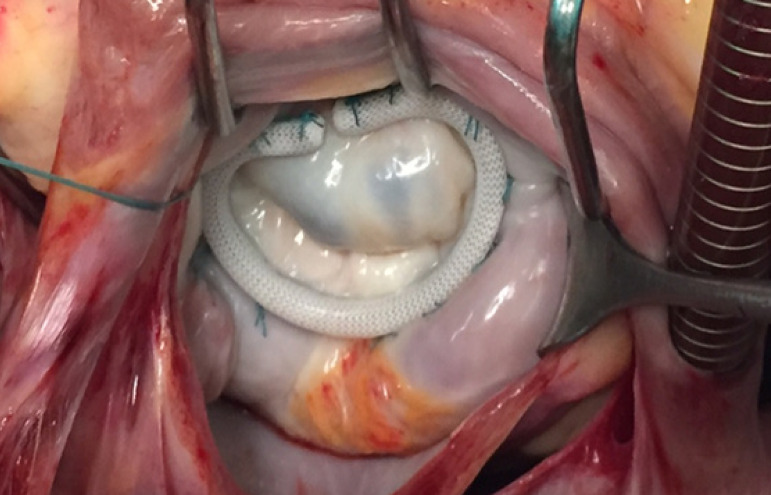
Rheumatic mitral valve repaired by PTFE chordae in the anterior leaflet (note the jet lesions behind the posterior annulus, hallmark of anterior leaflet prolapse) and complete rigid ring annuloplasty.

The use of a ring for the annuloplasty has been considered essential in the surgical correction of rheumatic mitral regurgitation (Figure 3). As an alternative to the complete, rigid ring, other types and shapes of rings and bands, complete or incomplete, have been used, pioneered by the Duran flexible, incomplete ring. In my opinion, based also on the experience of others, a complete ring is essential in rheumatic mitral regurgitation, not only to narrow de annulus but also to increase the coaptation between the anterior and posterior leaflets, best achieved by a rigid or semi-rigid ring.

In very young and small patients, with short leaflets, extension by autologous (or heterologous?) pericardium, fresh or treated with glutaraldehyde, which increases the coaptation area, has been used by some surgeons with promising results^[23]^. This technique also permits the use of a larger ring that allows better adaptation to the patient’s growth. Other techniques, such as resection and fenestration of thickened and retracted chordae, and leaflet thinning, to facilitate mobility, may complement the two basic surgical maneuvers.

Despite these technical advances, rheumatic valve repair remains challenging^[24,25]^. It requires experienced surgeons, but experience only comes with practice. Hence the encouragement for cardiac surgeons to learn and pursue mitral repair in these patients, having in mind the proven benefits by comparison with valve replacement. I, as well as others, have consistently received encouraging feedback from surgeons and residents who assisted to tutorials and wet labs or live operating room demonstrations, with a positive impact on their own surgical activity. Some have initiated and maintained mitral valve repair programs where they did not exist before.

### Access to Surgery. Role of Humanitarian Medicine

**Fig. 4:**
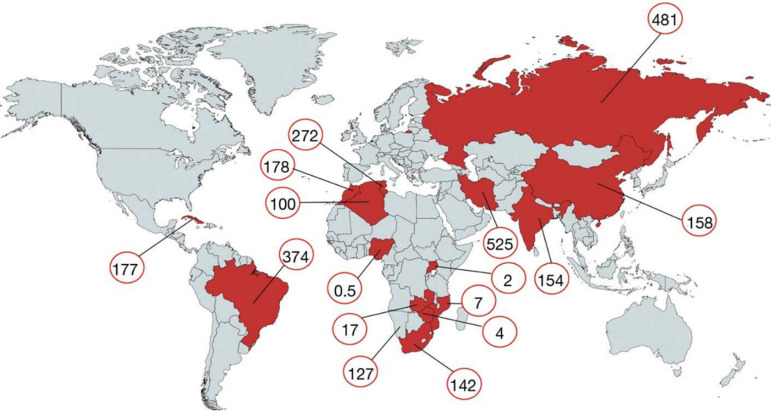
Number of cardiac operations (no. per million) performed in “rheumatic countries” (adapted from Zilla et al.^[4]^).

While surgery is easily accessible to patients in developed countries, the situation at LMICs is often dramatic, varying from countries where there is complete absence of surgical facilities to those where a single or only a few facilities are absolutely unable to meet gigantic demands. This is the case of most sub-Saharan African countries, with the exception of South Africa, that, however, still has some weaknesses in this area (Figure 4). Also, there are wide differences between countries, even considering different population numbers and economic status. And this is a more political issue than just a question of national financial capacity. Nigeria has a gross domestic product (GDP) similar to those of the Maghreb countries, yet it performs 0,5 cardiac operations per million people versus 117 in the Maghreb region. To aggravate the problem, the fast-growing population, naturally increasing demand, make resources even scarcer.

Although recent government initiatives in African countries led to the establishment of local, independent cardiac surgical services, these centres still only cover less than 2% of the needs of their populations^[4]^. In this regard, India is a good example of a country where a large number of well-developed cardiac surgical facilities is still clearly insufficient to meet the needs of a population that now exceeds one billion. Besides, these patients only have access to public hospitals, which are now largely outnumbered by private institutions where only a few of these cases are operated on a humanitarian basis. Brazil is also such a good study case, still with important differences from region to region, with wide recognition that the national health system (SUS) is also insufficient to meet the demands of a fast-growing population.

Many foreign surgical teams conduct humanitarian missions in several of the least developed countries. In most cases, these efforts are disperse and sporadic in time, limited to single or rare missions yearly, with few patients benefited each time^[26]^. They are just a “drop of water in the ocean”of needs. In some cases, however, these effort missions led to the establishment of local teams that now work independently and, in some cases, outperform the foreign teams that are still visiting. I was fortunate to participate (I still do) in one such experience in Mozambique, where a project developed by the generic Chains of Hope (French, English and Portuguese) created the Maputo Heart Institute in 2001, which has since become a locally prestigious hospital unit. Another example is the Institute of Cardiology in Abidjan, Abidjan, Ivory Coast, where a local team has now been operating for 35 years^[27]^. Similar and much larger institutions were developed in Vietnam and Cambodia, by French teams, and in the Aswan Heart Centre by Magdi Yacoub’s group. Unfortunately, these are rare exceptions that only confirm the rule.

## Conclusion

Until the incidence of RF is controlled, the demands for surgery for RHD are still very far from being met by the current facilities in most LMICs. Even in countries in this group that are at a higher level of development, such as China, India and Brasil, there is a pressing need for upgrading the national systems of health. External help, such as that of humanitarian missions, is always welcome and in some cases has succeeded in generating independent local units, but are far from solving the problem. The original idea certainly had merit, but “the model appears to be suboptimal for skill transfer and needs to be reshaped”^[28]^. In this regard, there have recently been calls in Africa for transnational shared efforts to improve the local conditions and provision of health care for all, as called for by the Cape Town Declaration, and the Abidjan Declaration for open-heart surgery development and financing in Sub-Saharan Africa^[29,30]^.

**Table:** 

**Author’s Roles & Responsibilities**	
MJA	Substantial contributions to the conception or design of the work; or the acquisition, analysis, or interpretation of data for the work; drafting the work or revising it critically for important intellectual content; agreement to be accountable for all aspects of the work in ensuring that questions related to the accuracy or integrity of any part of the work are appropriately investigated and resolved; final approval of the version to be published
